# The Efficacy of *V. odorata* Extract in the Treatment of Insomnia: A Systematic Review and Meta-Analysis

**DOI:** 10.3389/fneur.2022.730311

**Published:** 2022-06-06

**Authors:** Shuangfeng Huang, Qianqian Huang, Zhongbao Zhou, Junliang Zhang, Yan Zhan, Zhigang Liang

**Affiliations:** ^1^The Second Clinical Medical College, Binzhou Medical University, Yantai, China; ^2^Department of Gastroenterology, People's Hospital of Jimo District, Qingdao, China; ^3^Beijing TianTan Hospital, Capital Medical University, Beijing, China; ^4^Department of Neurology, The Affiliated Yantai Yuhuangding Hospital of Qingdao University, Yantai, China; ^5^Department of Neurology, Yantai Affiliated Hospital of Binzhou Medical University, The Second Clinical Medical College of Binzhou Medical University, Yantai, China

**Keywords:** systematic review and meta-analysis, *Viola* extract, insomnia, Pittsburgh Sleep Quality Index, insomnia severity index

## Abstract

**Aim:**

This systematic review and meta-analysis was performed to assess the effect of *Viola odorata* (*V. odorata*) extract in the treatment of insomnia.

**Methods:**

PubMed, EMBASE, and Cochrane Library databases, as well as references of related articles, were searched. Finally, four articles with five clinical trials including 224 patients were included in the analysis.

**Results:**

The obtained results indicated a greater improvement in total PSQI scores (MD,−4.67; *P* = 0.0002), subjective sleep quality score (MD,−0.91; *P* = 0.003), sleep duration score (MD,−0.77; *P* < 0.00001), and ISI score (MD,−6.30; *P* = 0.009) in the Viola extract group compared with the placebo group. However, the Viola extract group did not significantly differ in sleep latency (MD,−0.85; *P* = 0.08), habitual sleep efficiency (MD,−0.61; *P* = 0.21), sleep disturbances (MD,−0.36; *P* = 0.11), and daytime dysfunction (MD,−0.94; *P* = 0.12) from the placebo group.

**Conclusions:**

*Viola* extract supplement led to a greater improvement in total PSQI scores, subjective sleep quality score, sleep duration score, and ISI score compared with the placebo group of patients with chronic insomnia.

## Introduction

Insomnia is a highly prevalent sleep disorder, affecting ~10–30% of the population worldwide (1). Insomnia is characterized by chronic dissatisfaction with sleep that is associated with difficulty initiating or maintaining sleep, frequent awakenings during the night along with difficulty in returning to sleep, and/or awakening earlier in the morning than desired, lasting for at least three months and occurring at least three times per week (2, 3). This consequently results in some form of daytime function impairment. Insomnia presents a significant economic burden for patients, health services, and societies, costing an estimated >$100 billion per annum due to its impact on absenteeism and worker productivity (4). Available treatments for insomnia include pharmacological and non-pharmacological therapies. Currently available pharmaceutical therapies for sleep disorders have been associated with potential side effects such as tolerance and dependence, thus making it is necessary to identify alternative treatment approaches (5). Recently, there has been an increase in the use of herbal medicines as complementary and alternative treatments for insomnia, as herbal products are readily available and generally appear to be safe (6).

The effects of traditional Persian medicine (TPM) on treating insomnia have been investigated with a distinctive focus on *Viola odorata (V. odorata)*, which was found to exert sedative and hypnotic effects in animals or humans (7). *V. odorata*, which is commonly used in the form of violet oil, belongs to Violaceae family and is native to Iran (8). Trace amounts of melatonin (1.1 ± 0.1 ng/g) were detected in a 50% methanol extract of the violet flower by using the enzyme-linked immunosorbent assay (ELISA) method of quantification (9). Melatonin, which promotes sleep by activating the BK channel through a specific melatonin receptor and Gβλ (10), is a widely available non-prescription nutritional supplement for the treatment of sleep disorders with significantly fewer side effects (11). It is an active ingredient of *V. odorata* (9), which can explain its hypnotic and circadian-shifting effects (12). Over recent years, a growing number of studies have shown that treatment of insomnia with violet oil significantly improved sleep and insomnia severity index scores (13, 14). Additionally, it was more effective on sleep quality than sleep quantity (13). However, the findings on the efficacy of *V. odorata* in the treatment of insomnia have been controversial.

Thus, the aim of this study was to evaluate the effect of *V. odorata* extract in the treatment of insomnia via systematic review and a meta-analysis of clinical studies.

## Methods

### Protocol

The preferred reporting items of the systematic review and meta-analysis (PRISMA) guidelines were used as the methodology in this meta-analysis (15).

### Literature Sources and Retrieval Strategies

To identify published and unpublished trials, PubMed (until Oct 2021), EMBASE (until Oct 2021), Cochrane Library databases (until Oct 2021), and original references of the included studies were searched to evaluate the effect of *V. odorata* extract in the treatment of chronic insomnia. The search terms were as follows: {[(“viola” OR “viola” OR “violas”) AND “odorata”] OR [(“viola” OR “viola” OR “violet” OR “violets”) AND “oil”]} AND (“insomnia s” OR “sleep initiation and maintenance disorders”) OR “sleep initiation and maintenance disorders” OR “insomnia” OR “insomnias” OR “sleep initiation and maintenance disorders” OR (“chronic” AND “insomnia”) OR (“chronic insomnia”) OR (“sleep wake disorders” OR “sleep wake disorders” OR “sleep disorders”) OR (“sleep wake disorders” OR “sleep wake disorders”). There were no language restrictions on the inclusion of articles, and duplicate studies were excluded. Four reviewers independently selected RCTs (HS, ZJ, HQ, and ZZ). Three reviewers (HS, ZZ, and ZY) performed data extraction and two reviewers (HQ and LZ) performed data review. If there was a dispute, it was assessed by the third researcher.

### Inclusion and Exclusion Criteria

Inclusion criteria were: (a) *Viola* extract for treating insomnia was evaluated; (b) articles that offered related data and full-text content; (c) studies that provided accurate data mainly including the number of subjects and the valuable results of indexes; (d) clinical studies.

Exclusion criteria were: non clinical studies, including abstract, review, or comment; animal experiment; studies with incomplete data. Criteria for considering studies for the review were based on PICOS structure (Table 1).

**Table 1:** Criteria for included studies based on PICOS Structure.

	**Population**	**Intervention**	**Comparator**	**Outcomes**	**Study designs**
Inclusion criteria	Insomnia as defined by DSM-V, DSM-IV-TR, ICSD-2.	Viola extract.	Placebo.	Primary endpoint: PSQI global score; Secondary endpoints: PSQI component score and ISI score.	Clinical research.
Exclusion criteria	Unwillingness to continue cooperation with any reason; severe drug allergy; history of severe allergy to plants, and allergic rhinitis; pharmacologic or non-pharmacologic treatment for insomnia in the last few weeks, diagnosed with hemorrhagic disease and serious physical disorders, pregnancy, and breastfeeding, et.	Other therapy.	Other therapy.	Qualitative outcomes such as patient feelings; inadequate indicators.	Letters, comments, reviews, and animal experiment.

*PICOS, Population, Intervention, Comparator, Outcomes, and Study Designs; DSM-V, Diagnostic and Statistical Manual of Mental Disorders-V; DSM-IV-TR, Diagnostic and Statistical Manual of Mental Disorders, Fourth Edition, Text Revision; ICSD-2, The International Classification of Sleep; PSQI, Pittsburgh Sleep Quality Index; ISI, Insomnia Severity Index*.

### Quality of Assessment

The quality of studies was accessed by using Cochrane Handbook. The study was classified according to the Cochrane Handbook for Systematic Reviews of Interventions v.5.3.0. Three quality classification criteria were as follows: (+) study was considered as having a low possibility of bias for conforming to almost all quality criteria; (?) study was counted as having a secondary probability for fulfilling partial quality criteria or indistinct; (-) study was considered as having a high possibility of bias for conforming to bare quality criteria.

### Data Extraction

Two researchers read all articles and extracted the following data: (a) characteristics of study; (b) name of authors; (c) study design and sample size; (d) interventions among the groups; (e) route of the administration; (f) evaluation index, such as the total Pittsburgh Sleep Quality Index (PSQI) score, the PSQI scores of its components: subjective sleep quality, habitual sleep efficiency, sleep disturbances, daytime dysfunction, sleep latency, sleep duration, and Insomnia Severity Index (ISI) score. The primary outcome was PSQI global score, and a lower global score reflects a better quality of sleep. Data on secondary outcomes were PSQI component score and ISI score. Finally, all the extracted data were checked by another author. This research did not require ethical consent.

### Statistical Analysis

Rev man 5.4.0 was used for the analysis and integration of data. Mean difference (MD) with 95% confidence intervals (CI) was applied to evaluate continuous index, and odds ratio (OR) with 95%CI was applied to evaluate dichotomous index. If the result was *p*-value > 0.05, the fixed-effect model was used for analysis. *I*^2^ reflected the ratio of heterogeneity across trials. The random-effect model was adopted when the index showed *p* < 0.05 or *I*^2^ > 50%. The index with *p* < 0.05 was considered as statistically significant. Due to the small number of studies included, our study did not conduct subgroup analysis.

## Results

### Basic Characteristics and Search Process

**Figure 1:**
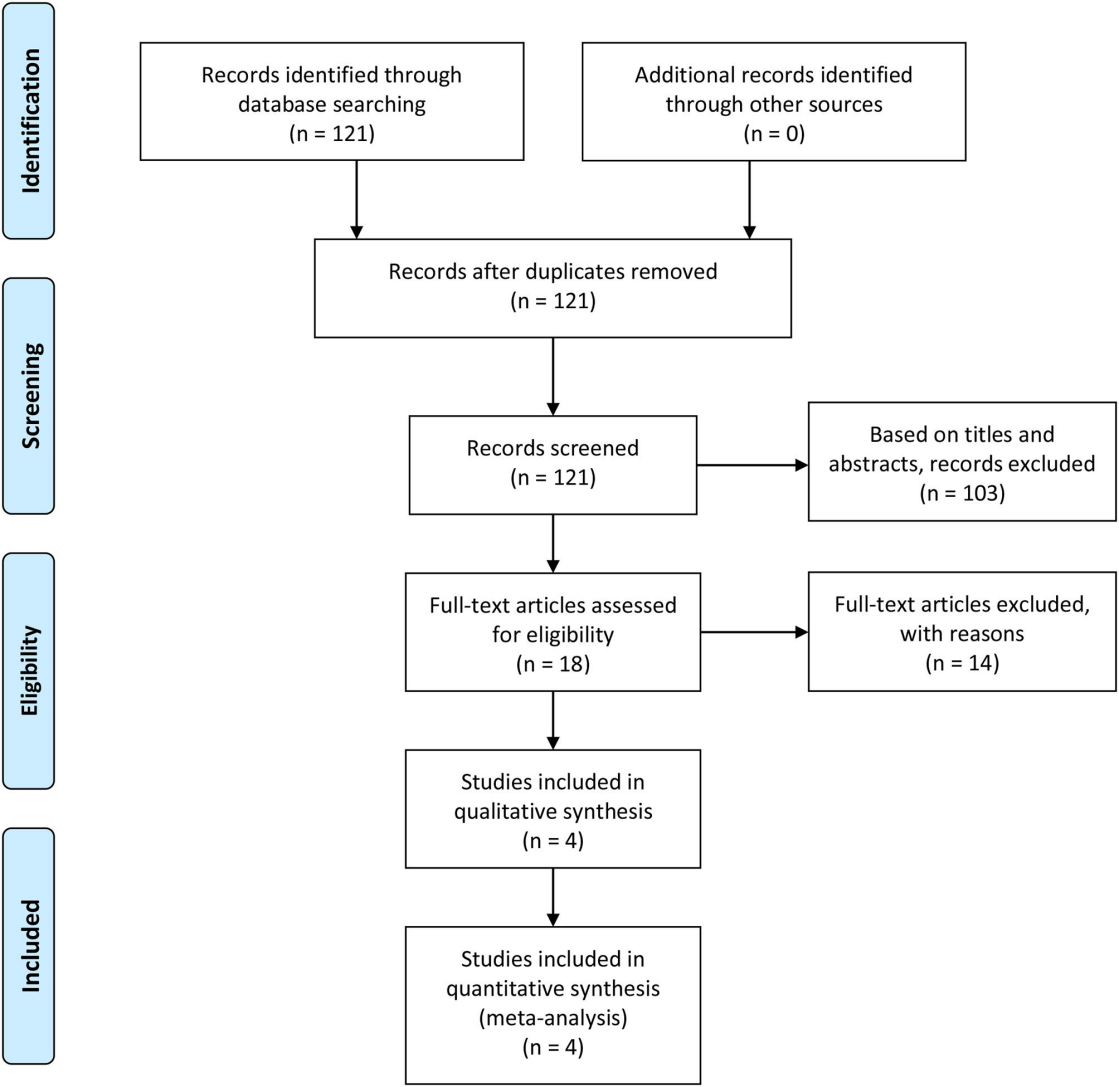
The flowchart of selection PRISMA. RCTs, randomized controlled trials.

We searched 121 studies in databases. Based on the inclusion criteria and exclusion criteria, 103 studies were deleted. Due to the lack of relevant information, 14 studies were excluded. The final 4 articles (7, 13, 14, 16) with 5 clinical trials met our inclusion criteria. Figure 1 shows a detailed selection PRISMA flow chart. Table 2 presents the basic characteristics of each study.

**Table 2:** The details of individual study.

**References**	**Study design**	**Treatment**	**Dosage**	**Sample size**	**Route of administration**	**Treatment cycle**	**Inclusion criteria**	**Calculation of sample size**
Shayesteh et al. (a) (14)	Double-blind randomized placebo-controlled trial	Viola odorata syrup; Placebo	5 ml/12h; 5 ml/12h;	20/20	Oral administration	4 weeks	16 to 50 years old, mild-to-moderate depression or OCD, insomnia symptoms confirmed based on the PSQI, and taking selective serotonin reuptake inhibitors for at least 1 month.	Yes
Shayesteh et al. (b) (14)	Double-blind randomized placebo-controlled trial	Viola odorata syrup; Placebo	5 ml/12h; 5 ml/12h;	23/20	Oral administration	4 weeks	16 to 50 years old, mild-to-moderate depression or OCD, insomnia symptoms confirmed based on the PSQI, and taking selective serotonin reuptake inhibitors for at least 1 month.	Yes
Taherzadeh et al. (16)	Double-blind randomized placebo-controlled trial	Herbal oil^a^; Placebo	2drops / every noon and evening; 2drops / every noon and evening;	25/25	Intranasal administration	8 weeks	18 to 40 years old of either sex; met the ICSD-2 criteria for primary chronic insomnia; primary sleep complaints for at least 1 month; no drugs abuse; and no parallel usage of spironolactone.	Yes
Feyzabadi et al. (7)	Pretest-posttest, cohort study	Violet oil	2drops (66mg)/daily;	50	Intranasal administration	4 weeks	16 to 50 years old, and meeting the defined criteria for primary insomnia according to DSM-IV-TR for at least three nights a week in at least 6-months period.	No
Feyzabadi et al. (13)	Double-blind randomized placebo-controlled trial	Violet oil; Placebo;	3drops^b^/daily; 3drops^b^/daily;	22/19	Intranasal administration	1 month	18 to 60 years old; having the pre-defined conditions of insomnia as DSM-V-TR specifies; lack of physical diseases, mental illnesses; lack of sleep disorders like sleep apnea; lack of drug and non-drug treatments for insomnia; lack of a job with a night shift.	No

*OCD, Obsessive ompulsive disorder; PSQI, Pittsburgh Sleep Quality Index; ICSD-2, The International Classification of Sleep Disorders; DSM-IV-TR, Diagnostic and Statistical Manual of Mental Disorders, Fourth Edition, Text Revision. ^a^A combination of violet oil, saffron oil, and lettuce 32 seeds oil. ^b^Each drop weighed 33 mg*.

### The Risk of Bias

**Figure 2:**
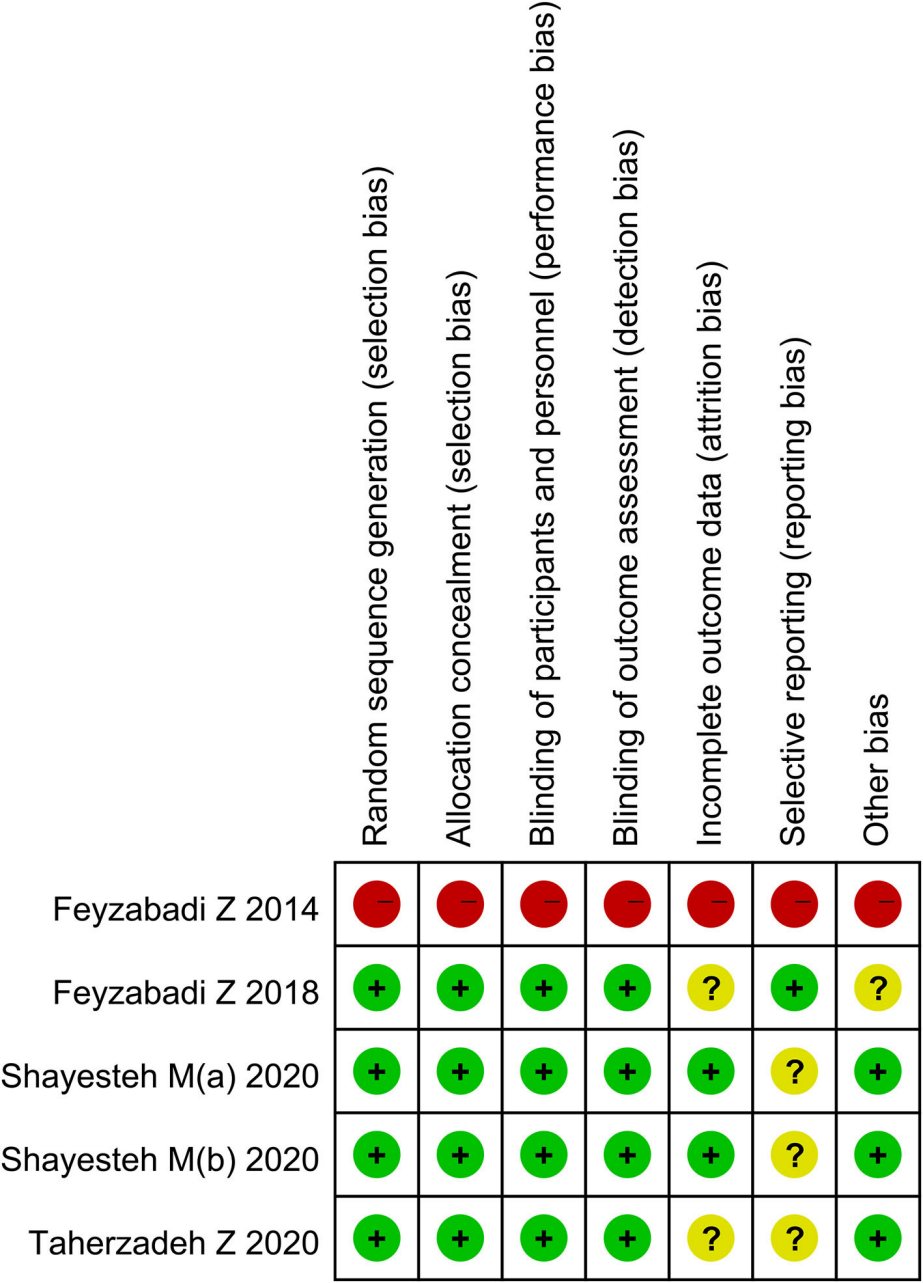
The risk of bias summary.

**Figure 3:**
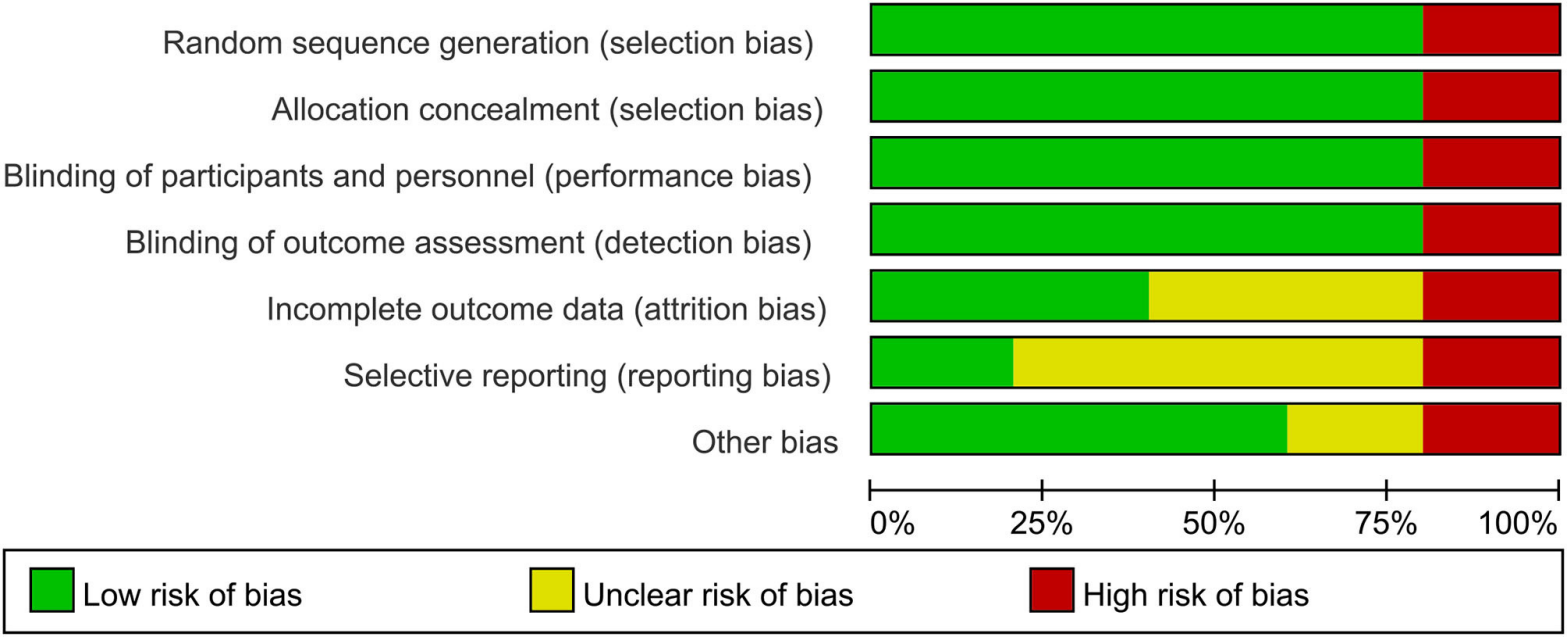
The risk of bias graph.

Three included articles were randomized controlled trials (RCTs); however, not all of the studies specified the protocol of randomization. Also, two studies described the calculation of sample size. Another included study was the pretest-posttest cohort study (Table 2). The outcomes of quality assessment are presented in Figures 2, 3. The plot was highly symmetrical and no evidence of bias was found (Supplementary Material).

### PSQI Global Scores

**Figure 4:**
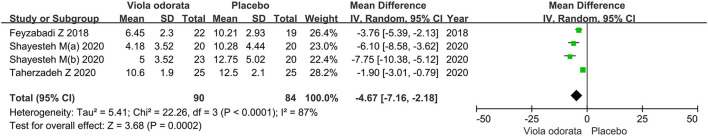
Forest plots showing results in total PSQI scores. PSQI, pittsburgh sleep quality index; SD, standard deviation; IV, inverse variance; df, degrees of freedom.

The analysis of PSQI global score was supplied by four studies including 174 patients, which showed high heterogeneity among the identified trials (*P* < 0.0001; *I*^2^ = 87%). Forest plots were drawn with an MD of−4.67 and 95% CI of−7.16 to−2.18 (*p* = 0.0002), which indicated that *Viola* extract caused a greater improvement of PSQI global scores in chronic insomnia compared with placebo (Figure 4).

### PSQI Component Score

The analysis of the PSQI component score was supplied by three trials, including 133 patients. Six of seven component scores (i.e., subjective sleep quality, sleep latency, sleep duration, habitual sleep efficiency, sleep disturbances, daytime dysfunction) were summed to obtain a PSQI global score in this study.

#### Subjective Sleep Quality Score

**Figure 5:**
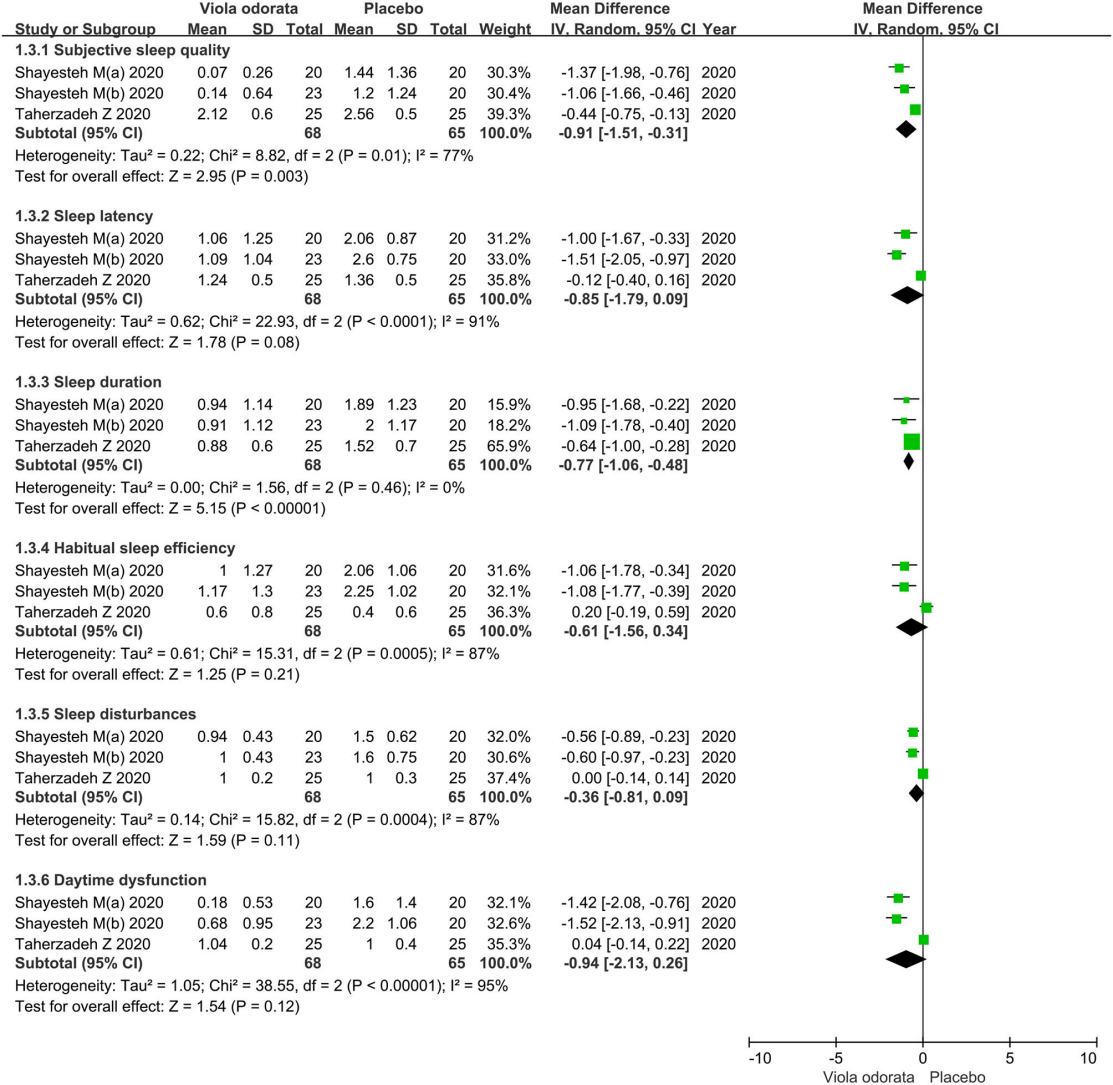
Forest plots showing results in PSQI component score. PSQI, pittsburgh sleep quality index; SD, standard deviation; IV, inverse variance; df, degrees of freedom.

Forest plots were drawn with an MD of−0.91 and 95% CI of−1.51 to−0.31(*p* = 0.003), indicating that *Viola* extract led to a greater improvement in subjective sleep quality for chronic insomnia than placebo (Figure 5). Significant heterogeneity (*P* = 0.01; *I*^2^ = 77.0%) was detected in the Subjective sleep quality.

#### Sleep Latency Score

Forest plots were drawn with an MD of−0.85 and 95% CI of−1.79 to 0.09 (*p* = 0.08), indicating no statistical significance between *Viola* extract and placebo in improving sleep latency for chronic insomnia (Figure 5). Considerable heterogeneity (*P* < 0.0001; *I*^2^ = 91.0%) was detected in the Sleep latency.

#### Sleep Duration Score

Forest plots were drawn with an MD of−0.77 and 95% CI of−1.06 to−0.48 (*p* < 0.00001), which indicated that *Viola* extract caused a greater improvement in sleep duration for chronic insomnia than placebo (Figure 5). Mild heterogeneity (*P* = 0.46; *I*^2^ = 0%) was detected in the sleep duration.

#### The Habitual Sleep Efficiency Score

Forest plots were drawn with an MD of−0.61 and 95% CI of−1.56 to 0.34 (*p* = 0.21), which indicated there was no statistical significance between *Viola* extract and placebo in improving habitual sleep efficiency for chronic insomnia (Figure 5). Considerable heterogeneity (*P* = 0.0005; *I*^2^ = 87%) was detected in the habitual sleep efficiency.

#### Sleep Disturbances Score

Forest plots were drawn with an MD of−0.36 and 95% CI of−0.81 to 0.09 (*p* = 0.11), indicating no statistical significance between *Viola* extract and placebo in improving sleep disturbances for chronic insomnia (Figure 5). Considerable heterogeneity (*P* = 0.0004; *I*^2^ = 87%) was detected in the sleep disturbances.

#### Daytime Dysfunction Score

Forest plots were drawn with an MD of−0.94 and 95% CI of−2.13 to 0.26 (*p* = 0.12), indicating no statistical significance between *Viola* extract and placebo in improving daytime dysfunction chronic insomnia (Figure 5). Daytime dysfunction model showed high heterogeneity *p*-value < 0.00001 and *I*^2^ of 95%.

### ISI Score

**Figure 6:**
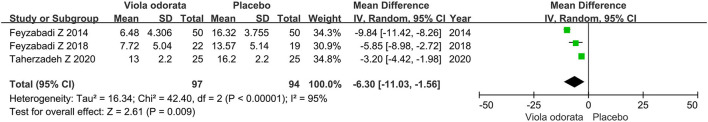
Forest plots showing results in ISI score. ISI, insomnia severity index; SD, standard deviation; IV, inverse variance; df, degrees of freedom.

The analysis of ISI score was supplied by two RCTs and one cohort study including 141 patients, which showed high heterogeneity *p*-value < 0.00001 and *I*^2^ of 95%. Forest plots were drawn with an MD of−6.30 and 95% CI of−11.03 to−1.56 (*p* = 0.009), indicating that *Viola* extract had a greater improvement in ISI score for chronic insomnia than placebo (Figure 6).

## Discussion

Insomnia is an important medical and social problem, with great severity and impact on wellbeing. Benzodiazepines are commonly prescribed to treat insomnia; nonetheless, they increase the probability of side effects such as amnesia, slowness, sleepiness, nervousness, forgetfulness, irritability, dizziness, and confusion (17, 18). TPM has been established as a valuable source of medicinal plants to treat insomnia for thousands of years (19). Based on TPM manuscripts, *Viola odorata* is one of the most cited and widely recommended medicinal herbs used to treat insomnia (7).

The aim of this meta-analysis was to confirm the role of *V. odorata* extract on sleep quality and sleep quantity in insomnia patients. The specific outcome variables included the sleep quality and the insomnia severity, which was measured by subjective measures (PSQI and ISI) and PSQI subjective sleep quality, habitual sleep efficiency, sleep disturbances, daytime dysfunction, sleep latency, sleep duration, and sleep quantity.

The obtained results demonstrated a greater improvement in total PSQI scores, subjective sleep quality score, sleep duration score, and ISI score in the *V. odorata* extract group compared with the placebo group. Yet, the *V. odorata* extract group did not show significant improvement in sleep latency, habitual sleep efficiency, sleep disturbances, and daytime dysfunction compared with the placebo group.

*V. odorata* is a kind of herbal medicine known for its hypnotic and sedative effects on headaches (20) and insomnia in ITM (21). TPM scholars considered that insomnia occurs due to dryness of brain temperament. According to their clinical approach, it was necessary to correct the brain dystemperament by herbal medicaments with wet temperament (21). *V. odorata* oil with cold and wet nature was considered useful in the management of insomnia. GABA is the major inhibitory neurotransmitter in the central nervous system. Ligands that activate GABA receptors possess sedative, hypnotic and anxiolytic effects. It has been shown that several natural flavonoids have an affinity for the benzodiazepine binding site of GABAA receptors (22). E.g., rutin, a flavonoid found in violet flowers, has been shown to possess sedative and anxiolytic-like effects through GABAergic neurotransmission (23). *Violet* oil is an almond or sesame oil-based extract of *Viola odorata*, which is available in nasal drops. Intranasal delivery provides an efficient, non-invasive method of bypassing the blood-brain barrier (BBB) in order to deliver therapeutic agents to the brain (24). This route of drug delivery has been presented as an important route of drug administration, especially for neurologic disorders (25). Three of the articles included in this study involved intranasal administration (7, 13, 16). Unfortunately, due to the small number of studies included in this study, we cannot conduct a subgroup analysis on the route of administration to verify.

The term insomnia is used to describe a wide range of alterations in the amount and type of sleep loss or perceived sleeplessness. Etiologies include insomnia provoked directly by intrinsic sleep disorders, extrinsic sleep disorders, or circadian rhythm irregularities. Overnight polysomnography is a standard tool in sleep medicine for evaluating sleep-related pathophysiology, sleep architecture, and sleep integrity (26). Actigraphy characterized by well circadian rhythms and circadian rhythm disorders also is an effect method of objective measurement for insomnia (27, 28). The PSQI and ISI questionnaires are short subjective tools to measure insomnia symptoms and consequences, and they are also used in this study. The PSQI has a high test-retest reliability and a good validity for patients with primary insomnia. This questionnaire consists of a global score ranging from 0 to 21, containing the following clinical components: sleep quality, delay in sleep onset, problems with sleep duration, sleep efficiency, sleep disorders, taking medication for sleep, and abnormalities in daily activities (29). The combination of scores from all components is used to obtain a global PSQI score as a measure of overall sleep quality. ISI is a reliable and valid instrument for detecting cases of insomnia in different populations, and it is also sensitive to treatment responses in clinical patients.

In their randomized, double-blind placebo-controlled trial, Feyzabadi et al. prescribed intranasal *Violet* oil drops for patients with insomnia for 1 month. These patients who did not receive any treatments were provided with sleep hygiene instructions. Moreover, the score of sleep quality was determined with the PSQI, and the score of the ISI significantly improved in the participants who reported no adverse effects (13). Previously, Feyzabadi et al. evaluated the hypnotic effect of *Violet* oil in 50 insomniac patients in a pretest-posttest study. All patients received *Violet* oil (two drops nightly, intranasal) for 1 month. The results showed that ISI scores decreased from 16.32 ± 3.755 at baseline to 12.58 ± 3.592 and 6.48 ± 4.306 after 15 and 30 days of treatment, respectively, (7). This suggests that the response to the treatment increases over time, unlike benzodiazepines that cause drug resistance after their consumption (7, 30). A randomized clinical trial of Hejazian et al., who investigated the effects of *V. odorata* oil nasal drops on sleep quality in the elderly using the PSQI, revealed significant improvements in sleep quality, increases in sleep duration, reductions in sleep disorders, and improvement of delays in falling asleep (16). These results are consistent with those obtained in a pilot study, which revealed that patients who had insomnia and depression or obsessive-compulsive disorder were not deprived of routine treatments for depression or obsessive-compulsive disorder (14). Therefore, use of the Viola extract preparation can be used as an alternative to current hypnotic medications. Moreover, this preparation can help reduce the dose of hypnotic drugs in insomniac patients (16).

There are some limitations in the present study that should be taken into consideration. The quality of the included studies was insufficient, mainly with reference to the study design, patient selection, result data extraction, diagnostic homogeneity, route of administration and dosage of treatment. In addition, the bias of selection factors and subjective factors may also affect the final results of this study. Therefore, the results of this study should be interpreted with caution. Given the subjective data collection method used in the present study, objective sleep measures such as polysomnography, actigraphy, observations, and other sensors are recommended that should be addressed by future researchers. The efficacy of the *Viola* extract in the treatment of chronic insomnia still needs to be verified through randomized controlled trials with larger sample sizes multiple arms.

## Conclusions

*V. odorata* extract supplement improves the total PSQI scores, subjective sleep quality score, sleep duration score, and ISI score compared with the placebo group in patients with insomnia.

## Data Availability Statement

The original contributions presented in the study are included in the article/Supplementary Material, further inquiries can be directed to the corresponding authors.

## Author Contributions

ZL and YZ conceptualized the study, supervised the study, and wrote—review and editing. SH, QH, and ZZ cured the data, investigated the study, contributed to resources, and wrote—original draft. SH, QH, ZZ, and JZ involved in formal analysis, contributed to methodology, and provided software. ZL administrated the project. All authors contributed to the article and approved the submitted version.

## Conflict of Interest

The authors declare that the research was conducted in the absence of any commercial or financial relationships that could be construed as a potential conflict of interest.

## Publisher's Note

All claims expressed in this article are solely those of the authors and do not necessarily represent those of their affiliated organizations, or those of the publisher, the editors and the reviewers. Any product that may be evaluated in this article, or claim that may be made by its manufacturer, is not guaranteed or endorsed by the publisher.

## Abbreviations

*V. odorata*: *Viola odorata*
PSQI: Pittsburgh Sleep Quality Index
ISI: Insomnia Severity Index
RCTs: Randomized Controlled Trials
TPM: traditional Persian medicine
MD: Mean difference
CI: Confidence Intervals
OR: Odds Ratio.
