# Supplementary figures and images for: The Efficacy of V. odorata Extract in the Treatment of Insomnia: A Systematic Review and Meta-Analysis
Source: Front Neurol. 2022 Jun 6;13:730311. doi: 10.3389/fneur.2022.730311 (PMC9207380; doi:10.3389/fneur.2022.730311)

Supplementary file: Publication bias.

PSQI global scores:

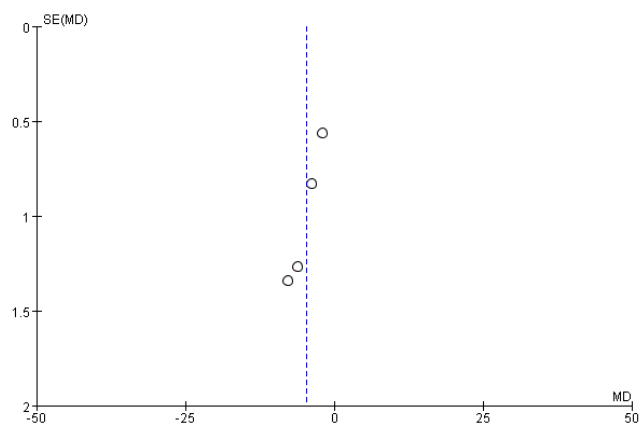

PSQI component score:

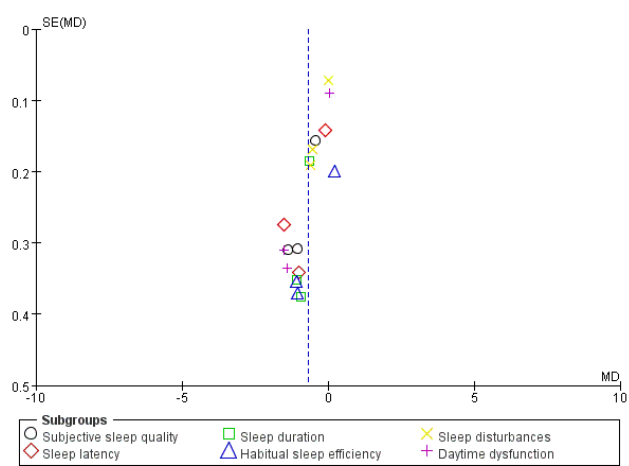

ISI score:

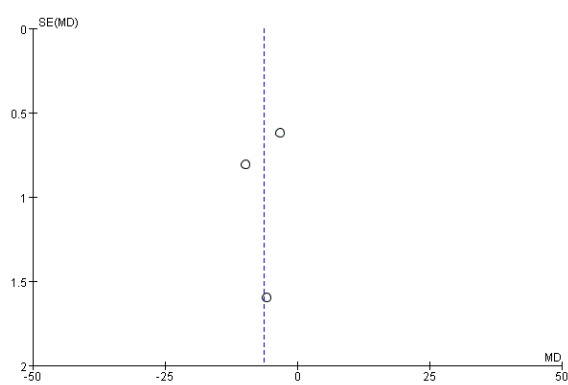

Supplement: Supplementary file 1 [file Data_Sheet_1.PDF]
